# A Case of Klippel-Trenaunay Syndrome Complicated by Group A Streptococcemia and Multiple Organ Failure

**DOI:** 10.7759/cureus.53910

**Published:** 2024-02-09

**Authors:** Ramaditya Srinivasmurthy, George Gilles, Tha Sok, Brian Chang

**Affiliations:** 1 Internal Medicine, Touro University Nevada, Henderson, USA; 2 Internal Medicine, Dignity Health St. Rose Dominican Hospital, Henderson, USA

**Keywords:** gas sepsis, group a strep sepsis, klippel, kts, klippel-trenaunay syndrome

## Abstract

Klippel-Trenaunay syndrome (KTS) is a rare congenital disorder defined as a triad of capillary malformation, venous malformation, and hypertrophy of soft tissue and bones, with or without lymphatic malformation. We report a case of a KTS patient with a hospital course complicated by Group A *Streptococcus* bacteremia and multiple organ failure. The 39-year-old female with KTS presented to the emergency department with a fever, tachycardia, hypotension, and profuse diarrhea for one week. Blood cultures grew Group A *Streptococcus* necessitating a multi-antibiotic regimen and intravenous immunoglobulins (IVIG). Secondary to septic shock, the patient's renal function continuously declined requiring eight rounds of hemodialysis. She was electively intubated due to worsening acute hypoxic respiratory failure. Chest X-rays demonstrated consolidation, pneumonitis, pleural embolism, and effusions. The patient also required eight units of packed RBC throughout her hospitalization. An underlying autoimmune etiology was suspected due to multiorgan involvement and abnormal blood smears, which was confirmed by an autoimmune panel. The patient ultimately was stabilized and was optimized for discharge. This case demonstrates the importance of a multidisciplinary approach in managing patients with KTS due to their associated lymphatic abnormalities that predispose them to severe infections.

## Introduction

Klippel-Trenaunay syndrome (KTS) is a rare congenital disorder that affects two to five in 100,000 people worldwide [1]. The etiology of KTS has been linked to somatic mutations in the *PIK3CA* gene [2], and is defined as a triad of capillary malformation, venous malformation, and hypertrophy of soft tissue and bones, with or without lymphatic malformation [1,3,4]. Varying presentations and severity of the vascular malformations lead to complications such as gastrointestinal bleeding, sepsis, or recurrent pulmonary embolism secondary to deep venous thrombosis [1]. Due to the complex multi-system involvement of KTS, management requires a multidisciplinary approach bringing together the experiences of different specialties [2]. We report the case of a KTS patient with a hospital course complicated by Group A streptococcemia and multi-organ failure. 

## Case presentation

A 39-year-old female, who was diagnosed with KTS in childhood, presented to the Emergency Department with a high fever of 104^o^F, tachycardia, hypotension, and profuse diarrhea for one week. Physical exam revealed unilateral enlarged lower leg, dilated tortuous varicosities, and arteriovenous malformation consistent with KTS. The patient’s initial blood culture grew gram-positive cocci. Together with her hemodynamic instability, positive blood cultures, and elevated lactic acid, the clinical presentation met the criteria for septic shock, so she was promptly started on an antibiotic regimen consisting of metronidazole, aztreonam, and daptomycin.

**Figure 1:**
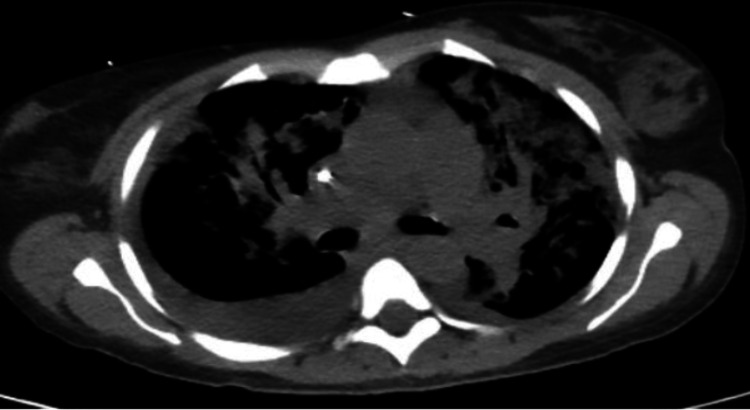
Chest CT demonstrating extensive bilateral airspace disease and pleural effusions

The Infectious Disease specialist was consulted when her repeated blood cultures grew Group A *Streptococcus* warranting a high suspicion for toxic shock syndrome. Based on the new findings, we transitioned her antibiotic regimen to a more narrow spectrum combination including meropenem, vancomycin, clindamycin, and intravenous immunoglobulins (IVIG). Secondary to septic shock, the patient's renal function continued to decline requiring eight rounds of hemodialysis. Her chest X-rays demonstrated multifactorial consolidation, pneumonitis, bilateral pleural embolism, and effusions (Figure 1).

The patient was electively intubated and ventilated due to increased oxygen demand and a worsening acute hypoxic respiratory failure. She was persistently anemic, requiring eight units of packed RBC given throughout her hospitalization. The patient was later found to have thrombocytopenia, and a peripheral blood smear revealed schistocytes, giant platelets, and target cells. Due to her multi-organ involvement, an underlying autoimmune etiology was suspected. Her autoimmune panel revealed she had a positive antinuclear antibody (ANA) panel with a speckled pattern, low C3 and C4 complement factors, and positive ANA HEp-2IgG. Ultimately, the patient became vitally stable and was optimized for discharge to continue her care at a large nonprofit academic medical center.

## Discussion

KTS was first discovered by Maurice Klippel and Paul Trenaunay, two French physicians in 1900 [5]. Initially referred to as angioosteohypertrophy syndrome, subsequent studies showcased the vast range of clinical symptoms and arterial and venous malformations that exist.

The severity of KTS depends upon the type of blood vessel dysplasia [1]. Type 1 is solely arterial dysplasia, type 2 is venous dysplasia (as seen in our patient), type 3a is arterial and venous dysplasia without an arterial-venous shunt, type 3b is arterial and venous dysplasia with a shunt, and type 4 consists of mixed angiodysplasias and tends to be the rarest form of the disease [1].

Patients with KTS have associated lymphatic abnormalities that predispose them to infections that lead to septic shock and/or bacteremia, as seen in this patient. Other organs involved in KTS include the gastrointestinal tract where bleeding can range from minor and persistent (as seen in our patient) to massive hemorrhage, the genitourinary tract causing persistent, painless hematuria, and skeletal abnormalities secondary to hypertrophy [1]. 

It is imperative to recognize the benefits of early detection and treatment of opportunistic infections in immunocompromised patients. Since the etiology of KTS is not entirely clear at the moment, the management of patients revolves around a conservative symptomatic treatment (such as compression stockings, leg elevation, and physiotherapy) and more importantly, a multidisciplinary team [5]. There are currently two targeted medical therapies for KTS: sirolimus, which is an mammalian target of rapamycin (mTOR) inhibitor, a downstream target of the mutated *PIK3CA* gene, and alepelisib, a *PIK3CA* inhibitor. However, both these treatment options are undergoing extensive clinical trials [6].

## Conclusions

Critically ill patients including KTS patients often require a multi-drug regimen to treat their diseases and mitigate further complications. Management of KTS includes a multidisciplinary approach, especially if the hospital course is complicated with streptococcemia leading to septic shock and multi-organ failure, therefore requiring the expertise of gastroenterologists, intensivists, infectious disease specialists, and nephrologists. Increasing education on rare diseases such as KTS along with prompt recognition of the necessary specialists will provide better clinical outcomes.
